# Inclusive and effective teaching of Nature-Based Social Prescribing for allied health professional students within digital environments

**DOI:** 10.3389/fpubh.2025.1694834

**Published:** 2025-12-15

**Authors:** S. Pywell, C. Wallace, S. Wallace, M. Davies

**Affiliations:** 1 Social Prescribing Unit, Institute of Creative Communities and Culture, School of Health, Social Work and Sport, University of Lancashire, Preston, United Kingdom; 2 Wales School of Social Prescribing, PRIME Centre Wales, University of South Wales, Wales, United Kingdom

**Keywords:** Social Prescribing, effective pedagogy, group concept mapping, nature, curriculum development, effective classroom practice, preferred learning environment, higher education inclusivity

## Abstract

**Introduction:**

Nature-based Social Prescribing is increasingly relevant to Allied Health Professionals in the UK, requiring educators to impart foundational knowledge on public health, health equity and nature connection. There is limited guidance on effective pedagogy and assessment strategies for inclusive and effective NbSP education, especially regarding contemporary approaches such as digital and mixed reality environments.

**Methods:**

This seminal mixed-methods integrative study employed Group Concept Mapping with thirty-eight stakeholders to explore perspectives on critical content ideas for NbSP training, pedagogical development and preferred learning environments for AHPs. Stakeholder input informed the identification of potential checklist items for curriculum design and effective classroom practice in-person and digitally.

**Results:**

Stakeholders emphasized forty out of one hundred statements as highly relevant for course design, content creation and the development of inclusive and effective teaching approaches for NbSP. Key findings highlighted the necessity of bringing students into nature and fostering direct nature connection alongside contributing to community organizations to understand more roles, in addition to traditional teaching methods. Opportunities to enhance higher education inclusivity were identified.

**Discussion:**

This study is the first to apply Group Concept Mapping to NbSP pedagogy for AHPs, offering initial insights for future curriculum design and assessment in this field. Further research is warranted to support educators in delivering inclusive and effective NbSP education, particularly in evolving digital and hybrid learning contexts.

## 1 Introduction

Teaching Nature-based Social Prescribing (NbSP) in Higher Education (HE) to Allied Health Professionals (AHPs) in the UK in an inclusive and effective way is fraught with complexities. Social Prescribing (SP), although notoriously ambiguous as a term and open to interpretation when exploring deeper meanings and models (1), can be defined at surface level as the connection of people with community assets by an “*appropriate person*” for health creation (2). NbSP can be defined as a “*cost effective*” SP intervention occurring within nature (3). Some (or all) of these components occur: nature connectedness (4), nature exposure (5), and nature for health with “*conferring environmental co-benefits, such as ecosystem restoration and biodiversity enhancement”* (6). NbSP activities include (yet are not restricted to): wilderness therapy, woodland programs, care farming, community gardening and activities in nature with a focus on reducing social isolation, loneliness and addressing health inequalities and mental health challenges (6–10). SP in the UK is known under the public health agenda connected to the government’s historic and current Long Term Plan (11, 12). Additionally, clients with long term (13) conditions including mental health issues (6, 14), and facing health equity challenges including the social determinants of health are identified as populations who can benefit from SP (15). However, ensuring AHPs understand SP works “*for whom, in what circumstances and why*” and their role in NbSP is part of learning to apply this approach in practice (16). This is important given the connections with SP and prevention, health promotion, reducing dependency forming medications (17) and positive changes through coaching (18).

A dearth of literature exists on how SP, and more specifically NbSP should be taught to AHPs resulting in a pedagogical gap. Yet, AHPs were identified as one professional cohort who could deliver on the SP agenda in the UK through four key roles: signposting to community assets, referring to a SP link worker (or receiving referrals) (19–21). Despite the international expansion of SP (22, 23), potential of NbSP to improve mental health outcomes as illustrated in a recent systematic review and meta-analysis (24) and previous actions to explore teaching SP within medical curricula and student placements (25–27), no explicit pedagogic framework exists for AHPs. SP can appear to lack extensive high quality quantitative research underpinning practice (8), leaving educators reliant on qualitative findings and experiential accounts. However, recent evidence developments support the use of NbSP and encourage “*advocacy*” for this public health intervention within integrated care systems (6, 8). Ongoing debate exists across HE as to the most appropriate way to teach within universities and the value and limitations of digital formats (28–31). It could also be argued uncoordinated systems exist where there is poor communication on the opportunities to embed NbSP in HE classrooms. Mechanisms to change national curriculum guidelines take time, as do the local changes educators make within the way they deliver and assess the curriculum within their HE establishment.

This seminal mixed-methods study explores these challenges from key stakeholder perspectives.

### 1.1 Teaching allied health professionals nature based social prescribing

A lack of teaching frameworks exists specifically on NbSP for AHPs impacting pedagogy and practice by educators in HE. SP placements for health and social care students were addressed by the Personalized Care Interprofessional Education (PerCIE) group (25). However, the specifics of how educators should teach NbSP in HE classrooms to AHPs in an inclusive and effective way has not been explored by any educational policy, framework, organization or author in the UK. Although support for students learning SP has been explored through the creation of local and national student SP networks (26), these are not mandatory for AHP students. The Council of Deans in 2021 highlighted SP should be taught, but this is not detailed enough as to include the specialism of NbSP (32). Lack of clarity remains where NbSP should be situated by educators within teaching topics. Despite significant weight being emphasized on AHPs learning public health, health promotion and prevention within the curriculum from national agendas (11, 12), there is no clear guidance on how NbSP should be taught, assessed and applied in practice to create positive change. Wider challenges including the lack of representation of SP and NbSP in National Institute of Care Excellence (NICE) guidelines could be an additional source interrupting positive action through understandings (33). Until SP is explicitly contained within all NICE guidelines, AHPs are likely to be aware of the SP agenda but not perceive to be able to act on it, given the severity of not following NICE guidelines in practice (33). One paper referred to adaptations Occupational Therapists may need to make to support the SP agenda including: use of language, working with SP link workers, and understanding community activity risks (34).

In the UK, the Health and Care Professions Council mandates AHPs to deliver on public health and prevention agendas, including SP, as illustrated in their “*Standards of proficiency: promoting public health and preventing ill-health*” (35). The AHP role in SP has been defined as important given the rise in health inequalities, extensive waiting lists within health and care services, and the skill set leaning into personalized, patient-centered approaches (19, 21, 33–38). AHPs, including OTs, were described as an “*essential partner*” in SP (34), and specific skills include delegating to, receiving referrals from and supervising SP Link Workers (21). Pre-registration AHP students need to understand SP and NbSP and the potential benefits for the people they work with in practice (32), however no guidance or consensus exists on how it should be taught within the curriculum. Although contemporary literature includes the effectiveness of teaching, inclusion and inclusivity is not always included (39).

AHPs need an awareness of and to actively action protection of climate and nature in their practice. This is seen within Sustainable Development Goals, National Health Service England (NHSE), Green AHP, Green SP and Net Zero agendas, although AHPs are not always aware of their role as illustrated in a recent systematic review (40). AHPs need to have the ability to critically explore the evidence and national (and international) agendas to begin to critically reflect on the impact of not supporting the individuals they work with to connect with nature through meaningful activity for health creation. Within certain professions, for example OT, debate remains on the role of the professional within SP (21). One paper highlighted the significance of the medicalized language of SP perhaps being used to bridge understandings of meaningful activity and therefore occupation in OT (34). Historically, AHP literature has highlighted the lack of understanding of SP and potential for impact within clinical services (38). Given the contemporary nature of NbSP, the extensive funding into NbSP across the UK (6), and specialist nature of the roles within it, it is reasonable to anticipate future developments in this field. All AHP groups created the need to update teaching for the purpose of preparing students for the real world of practice.

The aim of this seminal mixed-methods study is to explore the above challenges from key stakeholder perspectives. The study goals are to:

- Initially explore these challenges with a meaningfully sized sample of key stakeholders, to help explore definitions of inclusive pedagogy for UK AHPs in NbSP
- Determine key stakeholder-generated themes and identify issues of high priority and importance to enabling improved NbSP teaching of AHPs
- Identify areas of future research, in particular on extending this study’s findings to the diverse body of AHPs.

## 2 Methods

This explored the “who, what, where, when, why and how” of the pedagogy surrounding NbSP and what future pedagogy could hold. Data collection occurred in late 2024 and early 2025.

### 2.1 Ethical approval

In 2024 Ethical approval was obtained from the University of South Wales (LR 230270) and University of Lancashire (HEALTH 01109 EC). This approval was for Group Concept Mapping (GCM) of stakeholders’ perspectives on inclusive and effective teaching to AHPs of NbSP. The GCM tool “groupwisdom” (41) was used to facilitate remote coproduction on this preliminary scoping.

### 2.2 Sample size and attrition

38 participants were recruited in total across 3 workshops, email and social media recruitment. Participants in each workshop included a wide mix of stakeholders from education, research, policy creators, national organizations’, academia, practice, students, AHPs, and commissioning and community organizations. Brainstorming occurred across workshop 1 (conducted in late 2024 with *n* = 5 participants) and workshop 2 (conducted in early 2025 with *n* = 4 participants). Workshop 3 (*n* = 6 participants attended) and additional participants (*n* = 23) who took part by email (containing sorting and rating) occurred in May 2025 with each participant being tasked with sorting and rating. As sorting occurred as a task before rating, each workshop and task was delivered using sequential mixed methods, not concurrent. Ten participants were excluded after signing up due to completing less than 75% sorting and/or rating questionnaires. Transparent reporting of attrition is needed in GCM studies to address the validity of conclusions (42, 43).

Attrition bias is a threat to internal validity (44). Validity of conclusions were mitigated through transparency of attrition and potential impact on results, analysis of characteristics of those who dropped out, maximizing participation and reducing burden to participants (45). How the drop of *n* = 38 participants to *n* = 28 is reported affects the validity of conclusions and raises the question of acceptable dropout rates in GCM studies. Historically, not every GCM study has acknowledged attrition in depth, nor added to the debate, therefore there is limited discussion in the literature and a lack of consensus on an acceptable rate. Two studies identified the attrition rate as 48% for online GCM studies (45, 46). Yet, Lovegrove et al. (43) reported the potential for 10% attrition rate between GCM stages based on previous studies (47).

Despite the sample attrition, the sample is within pooled sample analysis size (45). Furthermore, it does raise the question how is every participant’s voice represented in this type of research? From the 10 participants who did not complete more than 75% of the questionnaire, 9 participants exited during sorting and 1 participant exited during rating. The attrition rate for this study is 26.32% overall, 47.4% in the sorting phase and 10% in the rating phase. These attrition rates align with existing literature of online attrition. No feedback was given from participants regarding non-completion and drop out therefore information is missing on participant perspectives of attrition. Attrition and overall sample size could be due to digital challenges, time taken to complete each full stage and intersectional challenges as outlined by Yu et al. (48). Further research into the potential for consensus on the acceptable attrition rate (including delineation of non-completion of questions or dropout rate) in GCM studies is required to support questions regarding representativeness of samples.

### 2.3 Group concept mapping process and groupwisdom tool

**Figure 1:**
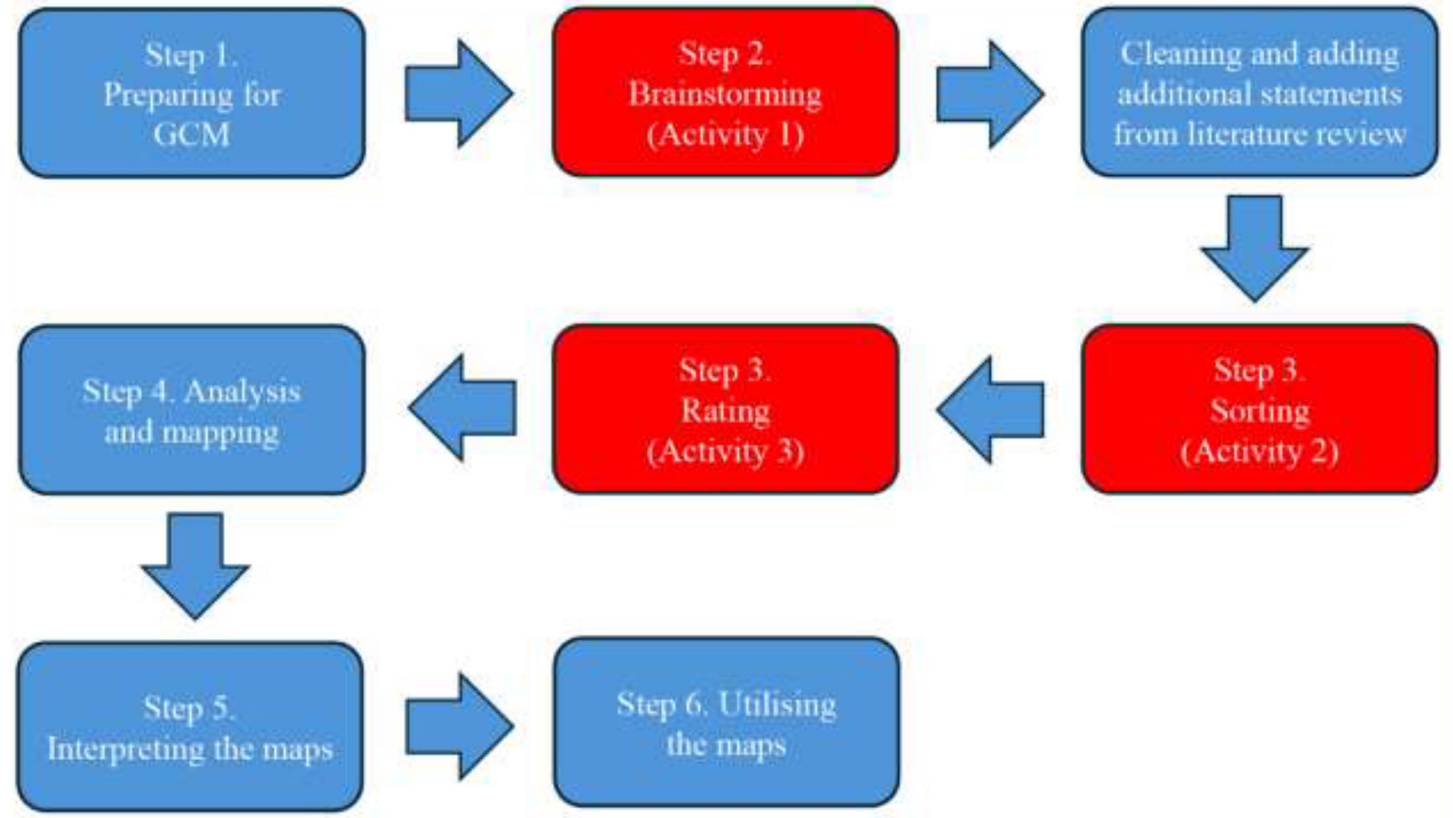
Flowchart of the GCM process. From Yu et al. (48), with permission.

Figure 1 outlines the GCM process, defined as a *“structured methodology for organizing the ideas of a group on any topic of interest and presenting those visually through a series of interrelated maps”* (49). GCM is the most appropriate method for effective and inclusive pedagogy exploration as it integrates participatory, quantitative and qualitative methods to capture diverse perspectives among varied stakeholders. GCM is unlike Delphi survey approaches in that the latter may constrain input or force consensus through repeated rounds and predetermined coding schemas (48, 50). Anonymous participation alongside independent completion of each stage allows participants to be freed from the potential constraints of groupthink or peer pressure as seen in Delphi studies (50–52). Thus GCM is highly appropriate for this research question focused on inclusivity and collaborative, emergent knowledge creation in pedagogy.

Historically, concept mapping has been used to explore stakeholder perspectives on topics including *“common vision,” “importance”* and *“priority”* of issues (49). Rosas and Kane (45) concluded GCM was a valid and reliable methodology following the pooled analysis of 69 concept mapping studies. The well established GCM research tool groupwisdom was used as a method when hosting the questions and participant answers due to its flexibility within historic SP research projects (2, 53). AHPs have used GCM for exploring the components of a quality improvement network (54).

In the field of SP, GCM and groupwisdom have been used to develop the SP Learning Needs Framework for practitioners in Wales (55). GCM was also used to identify and classify SP terminology in Wales (2) and in education GCM was used to define learning outcomes for an interdisciplinary module in medicine (50). GCM synthesizes stakeholder generated statements through statistical mapping and cluster analysis (2), yielding visual models of emergent themes highly suited for the inclusivity inherent in pedagogical exploration. Its participatory, mixed-method design ensures that participant voices are represented and facilitates iterative interpretation (45), supporting the collaborative aims foundational to inclusive pedagogy research.

### 2.4 Brainstorming

Entering the groupwisdom software (hosted by the University of South Wales) as an administrator, the lead researcher created the “*focus statement*” and “*focus prompt*” (41). The “*focus statement*” was “*generate short phrases or sentences that describe specific things that educators in HE should do to create effective and inclusive learning and teaching on NbSP education*.” The prompt: *“One specific thing educators in higher education need to do in order to effectively and inclusively teach Nature-Based Social Prescribing to the future health and care workforce is…”* was used with participants to create the initial brainstorming statements.

Nine participants generated 158 statements which were cleaned and merged into 100 statements following guidance (49). 100 statements are known to be the maximum recommended number of statements due to demands on participants therefore reducing statements by *“deleting synonyms or jointly grouping terms”* was actioned by the researcher (56). Statements were consolidated by “*splitting compound ideas*,” “*edited for clarity*,” “*choosing ideas*,” “*grouping ideas for reduction purposes*” and “*highlighting keywords in each statement*” for the purpose of combining or reducing statements to 100 (49). Each change to the statements was recorded in a Microsoft Excel document along with the reason for the change.

### 2.5 Sorting

Participants were asked to sort the 100 statements “in a way that made sense to them” (49). Participants received no additional verbal prompts. *N* = 10 participants completed sorting above the 75% threshold of inclusion (49). The sorting and rating sample size was initially set between 7 and 300, based on a pooled analysis by Rosas and Kane (45) of 69 studies exploring quality and rigor within concept mapping methodology (45). Although the optimum sample size was cited by one paper was 20–30, the paper did not discuss the potential of “information power” (57) and other peer-reviewed papers with smaller sample sizes of 5 participants (45).

For defining learning outcomes in HE, one study used a sample size of 9 participants in sorting and 7 for rating with the defence that participants were expert in the topic area and therefore a much smaller sample size was obtainable (50). They stated, “*the focused expertise required and the structure of the GCM process which aggregates individual judgements*.” Rosas and Ridings (58) stated *“sample sizes, sometimes as low as 7, are common and accepted in GCM especially when the goal is to capture diverse perspectives or when working with hard-to-reach populations*.” Given the diversity of stakeholders including practitioners, academics, researchers, people with lived experience, system leaders and topic specialists’ perspectives illustrated a mixed initial exploration of this topic area. This paper is also one of the seminal documents on use of GCM by educators to explore session content and pedagogic connections.

### 2.6 Rating

Statements were rated by participants on their importance and feasibility to action (49). *N* = 9 participants completed this stage above the 75% threshold of inclusion (rated over 75 statements). GCM software “*applies hierarchical cluster analysis using Ward’s Method to group statements that are closest to one another”* (41). The lead researcher checked these “scenarios” and the subsequent headings given by participants to each pile to identify the themes from the research data (41). A “Go Zone” was created by the software, illustrating participant responses with “high feasibility and high importance” (41, 49).

## 3 Results

### 3.1 Demographics

A diverse group of stakeholders was represented in this research. Participants stated their experience of NbSP was mostly from England 35.39% with some from Wales (5.08%) and the majority not answering the question (59.32%). This may not reflect the participants’ place of birth or current place of residence. Participants place of work included local authority, community voluntary or faith sector, educator in HE, SP Link Worker, AHP, person with lived experience, Healthcare or Social Care/work.

Most participants had 13–36 months experience where they had worked in/referred to/used SP. Theory and evidence of SP (22.03%), practice of SP (10.17%) and models of SP (6.78%) were the three areas participants stated they had the most knowledge about. When asked “What is the most effective way to teach NbSP to future health and social care students in your opinion” participants highlighted three top preferences. These included: “*in practice/practical*,” “*by experiencing a social prescription in nature*” and “*outside*.”

### 3.2 Stress value

The stress value in GCM is a statistic illustrating how close the visual map matches the way participants grouped and sorted statements during this exercise (41). Newstead et al. (2) (p. 9) stated “A stress value lower than the calculated average verifies that the participants sorted the statements in a similar manner, with lower stress values indicating greater reliability (45) (p. 10).” The stress value result was 0.3332, within the range of 0.21 and 0.37, illustrating a reliable result (2).

### 3.3 Brainstorming

The 100 statements are not in priority order, but rather an order of occurrence, i.e., as participants entered the statements into the software this is the order they appeared in Table 1. The statements were consolidated from 158 to 100 by removing duplicates and consolidating similar statements as outlined in the methodology section. Upon completion of the “brainstorming” phase, 100 statements from participants were created (Table 1). Results are illustrated across stress value, brainstorming statements, sorting, and rating.

**Table 1:** Statements from participants in ‘Brainstorming’ workshops 1 and 2, group concept mapping.

No.	Statement
1	Improving nature connection increases the amount of pro-environmental behaviors.
2	Having plants in your clinic room
3	A nature day, e.g., https://www.lancswt.org.uk/wild-wellbeing-day
4	Holistic health new toolkit
5	Examples of what good looks like in HE institutions
6	Universities’ environmental and net zero targets
7	Alternative method of teaching
8	Arts and nature
9	Ways for students to think about it from different perspectives
10	Students creating their own content for virtual environments
11	Value for money conversations on Universities’ fees and teaching in nature
12	Nature champions
13	Encouraging students to be outside in green spaces to see the true benefit to them
14	Nature connection and exposure with trees, e.g., sit down and truly look at a tree, identify the tree and truly feel the presence of nature around them
15	Simplifying nature connection
16	People might not have the opportunity to access nature, e.g., local park might be a space where anti-social behavior occurred
17	Widening participation and nature connectedness
18	How to engage people to educate on nature connection
19	VR is not a replacement or a complete alternative. It’s an addition.
20	VR as a stepping stone to eventually getting out into nature.
21	Through VR put yourself in the shoes of someone who does not use nature daily - empathy and reflection
22	Nature observation and spotting, e.g., see a bug
23	Coding nature
24	A dual approach to teaching students
25	Virtual meditation
26	Staff well-being: a VR booth to lie down on in a reclining chair and have a headset on.
27	Taking simulated patients with different health conditions out into nature as experiential learning
28	Experience the kind of fullness of nature
29	Five pathways to nature connection
30	Research that people need to experience it
31	A landscape or a space where people with impaired vision actually see it
32	Augmented reality to engage them in those activities to see what they are like, to see those spaces
33	Help for people to understand or navigate those spaces that we are trying to teach them about
34	New VR, e.g., NHSexpo
35	Nature parks
36	Explore students’ understandings of Nature Connectedness
37	Using a forest school approach links in to using nature to support a variety of learning needs
38	Understandings of how humans learn
39	Nature into the learning
40	Film footage of nature, i.e., BBC Nature films/listening to nature sounds - e.g. mindfulness minute (from Spring watch)
41	VR nature connection activities to initially engage students who have not experienced nature connection
42	VR as a stepping stone for students to scaffold learning
43	VR as a tool to support students practice the social aspect of communicating (listening and talking) with a VR patient
44	Forestry England digital nature resources for use in indoor settings
45	Previous research has shown that people are more likely to refer to GSP when they have experienced themselves
46	Taking your patients out on walking meetings
47	Relationship between higher education, environments and sustainability goals
48	Climate action policies, e.g., net zero and sustainability targets
49	Teaching in nature to improve learning outcomes
50	Poems and other kinds of artistic activities, which can “create” deep connections with nature even without having to be in an actual environment
51	Students to learn and then be teachers themselves
52	Opportunities for students to play in nature
53	Taking students into different environments and ask them to engage in nature
54	Taking children outside
55	Careers in environment education
56	Personal experience
57	Help for students to get connected to nature
58	Listening to nature
59	Nature pathways either through VR or through the physical experience of being in with nature.
60	Widening participation in HE
61	A range of VR role play scenarios where students talk with a VR patient- How a health professional goes through that what matters to you conversation with their patients, link worker sat in an office, home visit, benefits of nature connection if showing signs of postnatal depression
62	Indoor resources supporting nature connection, e.g., portable rock pool or multisensory rummage box for people in elderly homes
63	Challenges of accessing nature in 15 min
64	Having a 15-min break where you are as immersed in nature as you can possibly be in a medical setting
65	Setting up particular barriers a patient with a particular health condition might encounter and try examples of risk assessment with a simulated participant
66	Barriers: transport to the NbSP, wearing VR equipment
67	Green Social Prescribing
68	Reflections on how much you can experience it using VR, e.g., does it engage all the senses?
69	Examples of access requirements for people with disabilities and accessible nature spaces, how they may navigate certain natural space in certain activities, student experiencing some of these.
70	AR and similar sort of simulation spaces to create an environment that is not felt by the students
71	Experiences of nature-based activities
72	Exploration of student understandings on Nature Based Social Prescribing
73	A nature experience recreated in VR
74	VR headset in your clinic
75	Department for Education guidelines
76	Connecting students with nature
77	Some environments aren’t always conducive to learning for people with different needs
78	Engaging in it in their own way
79	Whole organization approaches
80	Ways to support engagement and understanding for carers
81	Safe learning environments, e.g., nature, VR, classroom
82	The importance of increased connection to nature for people from working class environments and areas of high deprivation
83	Observing something really little to connect to nature, e.g., birds’ behavior
84	Not take away the opportunities where people can go into nature as many more benefits than in a virtual setting
85	Nature sounds in a guided walk
86	Logistics of getting 50 to 100 students out to a care farm
87	Relating the learning to being outside
88	Connecting these concepts for many different students
89	Opportunities around alternatives with nature incorporated
90	Thinking about their well-being and how they keep themselves well
91	Peer-to-peer learning
92	Questions for students: So, do you like what you see? What do you feel? What do you experience? What can you see?
93	Urban spaces and nature connection
94	VR is completely different to being out in nature
95	A food growing space
96	An outdoor classroom works really well for students who struggle in a confined space of a classroom
97	Multiple methods of presenting this information
98	Confidence of being nature
99	Value of nature
100	Theory and evidence supporting practice, e.g., the NHS Long Term Plan, Biophilia theory, nature connection, nature exposure

### 3.4 Sorting

**Figure 2:**
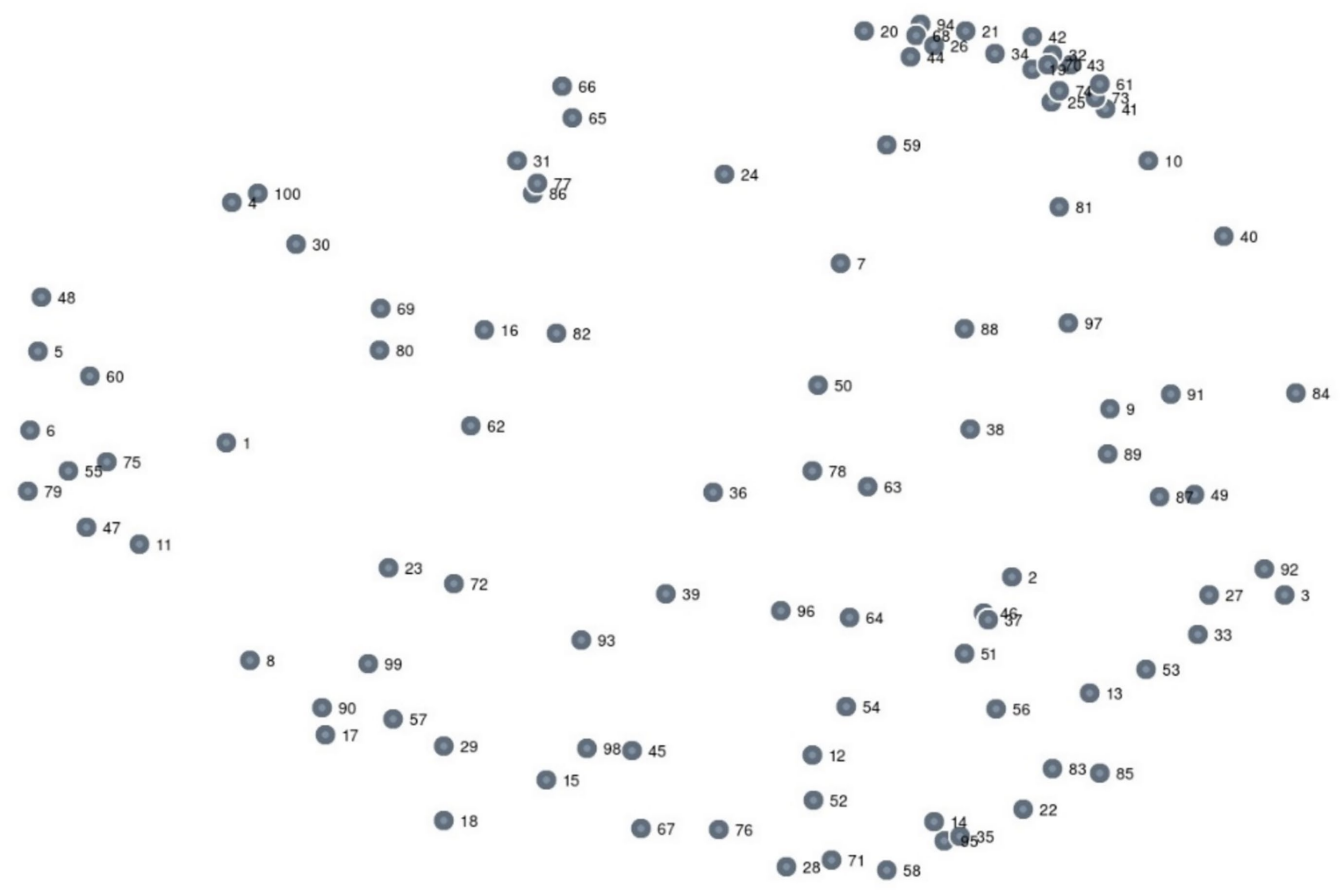
Point map.

The sorting “point map” is illustrated in Figure 2. Each statement from participants is listed in Table 1. The 100 points on the map represent the statements from participants where each point is an idea (56). The map shows how each idea is related (41). Where points are clustered together these are due to multiple participants stating similar ideas (46). This process is known as “multidirectional scaling” (41).

### 3.5 Rating

**Figure 3:**
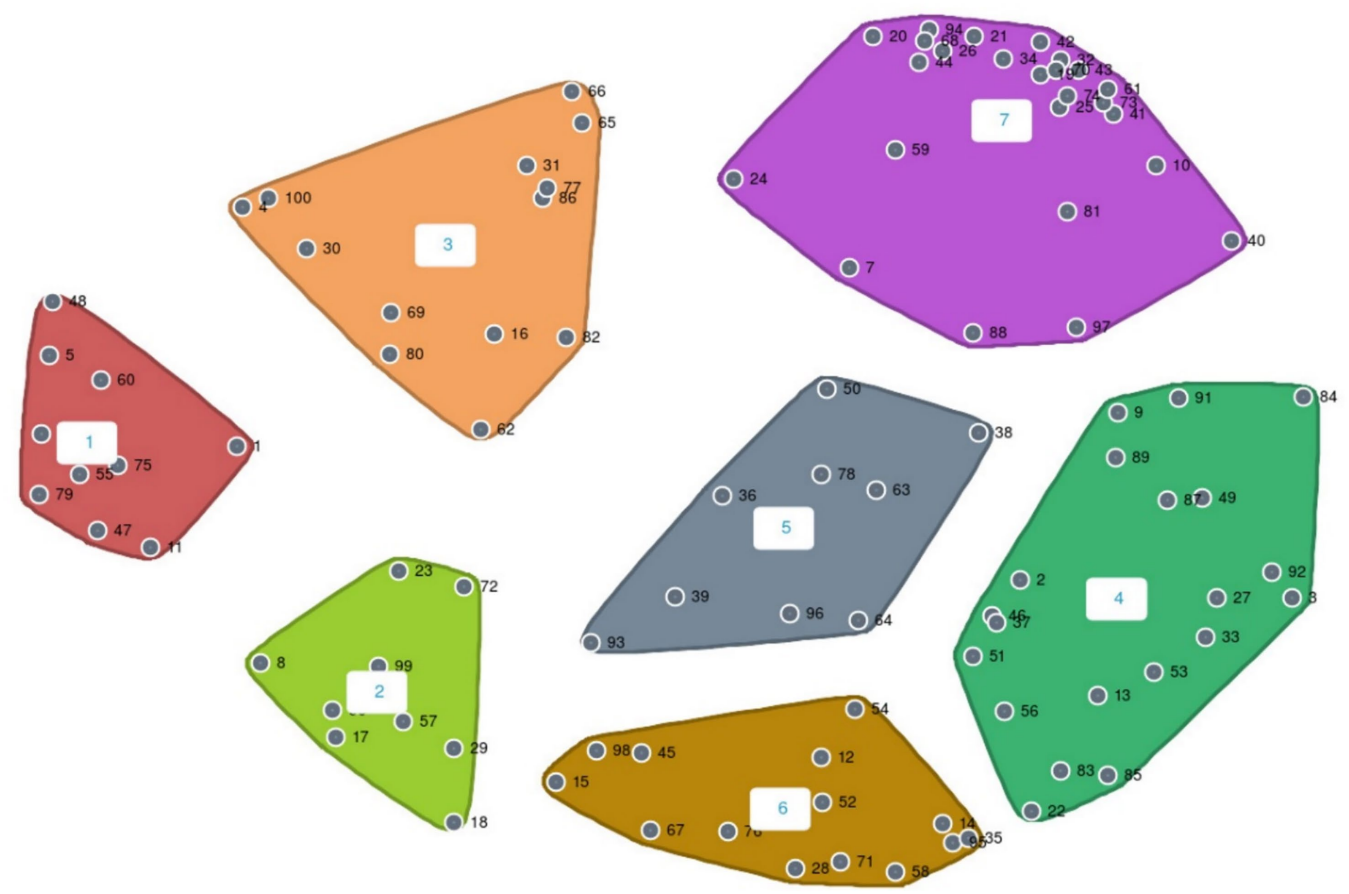
Cluster map.

Cluster maps illustrate “*where the boundaries are drawn around the points to create thematic clusters”* (41). The cluster map (Figure 3) of participant responses illustrates 7 key themes created by participants alongside Standard Deviation (SD) values: 1. Environmental careers (SD = 0.079), 2. Other benefits from this approach and learning (SD = 0.10), 3. Politics (SD = 0.15), 4. Educators should provide (SD = 0.096), 5. Nature as part of self-development and curriculum learning (SD = 0.063), 6. Experiencing nature to foster connectedness with nature (SD = 0.063), 7. Virtual Reality-based activities (SD = 0.14). Clusters with low SD values indicate the highest congruence (49). Three quotes are used from each section on the point map to illustrate participant results below.

*Environmental careers* were highlighted as important to encourage within AHP teaching (in the context of the social mobility of the people they work with and their own CPD aspirations). Examples from participants included “*widening participation,” “people might not have the opportunity to access nature,* e.g.*, local park might be a space where anti-social behavior occurred”* and *“ways to support engagement and understanding for carers*.”

Other benefits from this approach and learning included: “a nature experience recreated in VR”, learning “five pathways to nature connection” for personal and professional use and “arts and nature”.

*Political issues* impacting the inclusive and effective teaching of NbSP included: *“theory and evidence supporting practice,* e.g.*, the NHS Long Term Plan, Biophilia theory, nature connection, nature exposure*.”

Participants voiced educators should: *“not take away the opportunities where people can go into nature as many more benefits than in a virtual setting,” “using a forest school approach links in to using nature to support a* var*iety of learning needs*” and “*help for people to understand or navigate those spaces that we are trying to teach them about.”*

Nature was highlighted as part of self-development and curriculum learning through: “*having a 15-min break where you are as immersed in nature as you can possibly be in a medical setting,” “an outdoor classroom works really well for students who struggle in a confined space of a classroom,”* and for topics including “*urban spaces and nature connection.”*

Experiencing nature to foster connectedness with nature: *“experience the kind of fullness of nature,” “confidence of being nature*” and accessing “*nature parks*.”

VR-based activities suggested by participants included *“through Virtual Reality put yourself in the shoes of someone who does not use nature daily for empathy and reflection*,” “*Virtual Reality as a steppingstone for students to scaffold learning*” and *“Virtual Reality is not a replacement or a complete alternative. It’s an addition*.”

### 3.6 Pattern match

**Figure 4:**
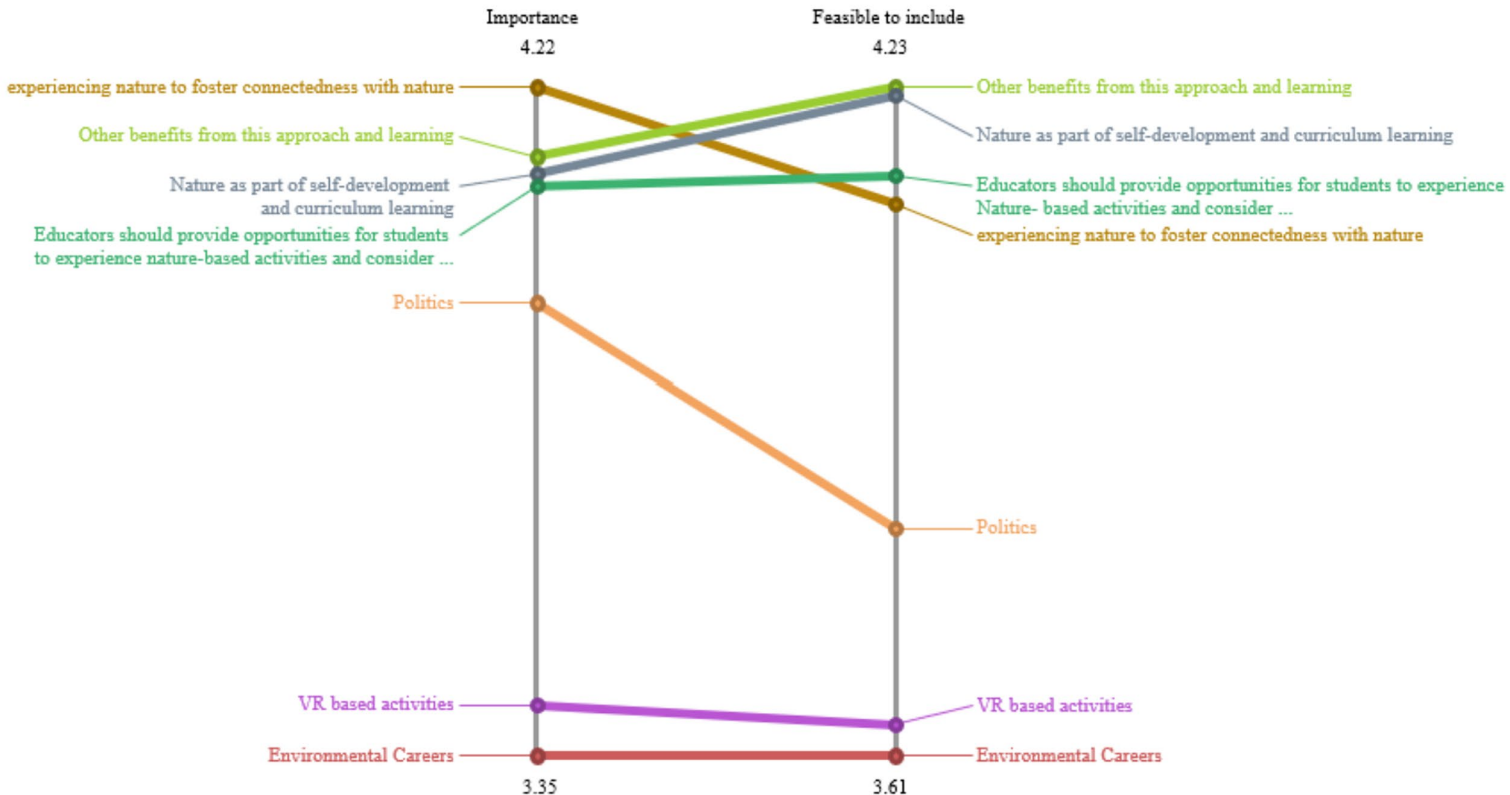
Pattern match.

Figure 4 illustrates the pattern match report results comparing cluster ratings for understanding and significance. The pattern match illustrated high Pearson correlation (r = 0.93) indicating higher agreement or congruence in perceptions of importance and feasibility results from participants (41). Interestingly, this group of participants rated experiencing nature to foster nature connectedness, other benefits and nature as part of self-development and curriculum learning as the most important actions for educators. Environmental careers and VR-based activities were rated by participants as being the least important and feasible to include.

Participants reported other benefits from this approach and learning, nature as part of self-development and curriculum learning as the most feasible to include. Despite participants completing the stages separately and not all participants completing every stage in GCM, there is harmony across several perspectives on the pattern match diagram shown in Figure 4.

### 3.7 Go zone analysis

**Figure 5:**
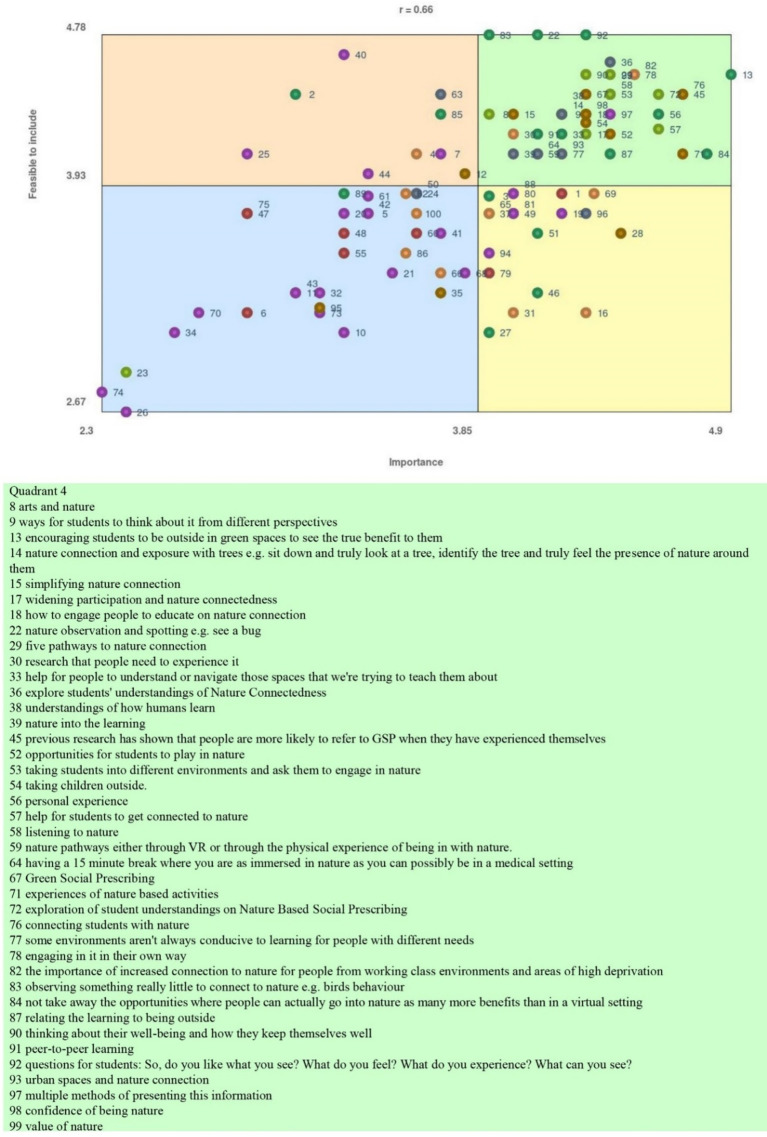
Go Zone analysis.

Key findings are represented in the “Go Zone” analysis based on participants sorting and rating the feasibility to include an importance of statements highlighted statements (Figure 5) of priority to them (41). Figure 5 shows the output of the Go Zone analysis and quadrant 4 statements (most important, most feasible), and Table 2 provides justification for statement inclusion in quadrant 4.

**Table 2:** Go Zone and quadrant 4 participant statements.

Focus prompt: “*When creating a virtual or digital environment which simulate nature and social prescribing for teaching health and social care students in higher education, I should include…”*		Importance scale	Feasible to include scale
#	Statements	Mean	Mean
8	Arts and nature	3.9000	4.3333
9	Ways for students to think about it from different perspectives	4.2000	4.3333
13	Encouraging students to be outside in green spaces to see the true benefit to them	4.9000	4.5556
14	Nature connection and exposure with trees, e.g., sit down and truly look at a tree, identify the tree and truly feel the presence of nature around them	4.2000	4.3333
15	Simplifying nature connection	4.0000	4.3333
17	Widening participation and nature connectedness	4.3000	4.2222
18	How to engage people to educate on nature connection	4.3000	4.3333
22	Nature observation and spotting, e.g., see a bug	4.1000	4.7778
29	Five pathways to nature connection	4.4000	4.5556
30	Research that people need to experience it	4.0000	4.2222
33	Help for people to understand or navigate those spaces that we are trying to teach them about	4.2000	4.2222
36	Explore students’ understandings of Nature Connectedness	4.4000	4.6250
38	Understandings of how humans learn	4.2000	4.3333
39	Nature into the learning	4.0000	4.1111
45	Previous research has shown that people are more likely to refer to Green Social Prescribing when they have experienced themselves	4.7000	4.4444
52	Opportunities for students to play in nature	4.4000	4.2222
53	Taking students into different environments and ask them to engage in nature	4.4000	4.4444
54	Taking children outside.	4.3000	4.2857
56	Personal experience	4.6000	4.3333
57	Help for students to get connected to nature	4.6000	4.2500
58	Listening to nature	4.4000	4.4444
59	Nature pathways either through VR or through the physical experience of being in with nature.	4.1000	4.1111
64	Having a 15-min break where you are as immersed in nature as you can possibly be in a medical setting	4.1000	4.1111
67	Green Social Prescribing	4.3000	4.4444
71	Experiences of nature-based activities	4.7000	4.1111
72	Exploration of student understandings on Nature Based Social Prescribing	4.6000	4.4444
76	Connecting students with nature	4.7000	4.4444
77	Some environments aren’t always conducive to learning for people with different needs	4.2000	4.1111
78	Engaging in it in their own way	4.5000	4.5556
82	The importance of increased connection to nature for people from working class environments and areas of high deprivation	4.5000	4.5556
83	Observing something little to connect to nature, e.g., birds’ behavior	3.9000	4.7778
84	Not take away the opportunities where people can go into nature as many more benefits than in a virtual setting	4.8000	4.1111
87	Relating the learning to being outside	4.4000	4.1111
90	Thinking about their well-being and how they keep themselves well	4.3000	4.5556
91	Peer-to-peer learning	4.1000	4.2222
92	Questions for students: so, do you like what you see? What do you feel? What do you experience? What can you see?	4.3000	4.7778
93	Urban spaces and nature connection	4.2000	4.1111
97	Multiple methods of presenting this information	4.4000	4.3333
98	Confidence of being nature	4.3000	4.3333
99	Value of nature	4.4000	4.4444

Go Zone results can be summarized under experiencing and connecting with nature (direct interaction and wellbeing), widening participant and educating for nature connectedness and deepening understanding and reflective learning about nature connection. Although these are not generalizable, these results are representative of the perspectives of the participants who took part in this study. These results highlight to future educators of NbSP potential areas of exploration within their teaching through reflection. Reflections could include what to teach, how to teach it in an inclusive and effective way, and why to teach this topic area to AHPs. Participant results could be used within course delivery, design and evaluation processes. The discussion explores the upper right quadrant results in more depth given these are the statements participants rated as most important.

## 4 Discussion

### 4.1 Criticality of AHP education incorporating awareness and action on NbSP

Contemporary evidence supports nature connection, nature exposure and nature for health for people prior to being referred into health services and after diagnosis (35, 59). The majority of participants’ statements across the Go Zone (Figure 5) and particularly in quadrant 4 connected to the criticality of teaching AHPs nature connection (7), nature connectedness (4), nature exposure (5), nature for health and nature for mental health in a way students can empower the people they work with (6). Participant responses indicated students need experiences connecting with nature in a simplified way, e.g., “*encouraging students to spend time outdoors in green spaces to recognize the benefits,” “relating learning activities to being outside and considering personal wellbeing*” and “*promoting nature connection and exposure – sitting with or observing a tree, identifying natural elements and truly feeling present*.” Numerous statements connected nature connection with positive health outcomes reinforcing public health agenda underpinning NbSP. Nature connection, exposure and nature for health were rated by participants as highly feasible and important in HE. Future research could explore more stakeholder perspectives and additional potential changes required to teach NbSP to AHPs.

Participants highlighted the benefits of reflective learning about nature connection to foster deeper understandings to impart to their clients. Although teaching in digital environments was acknowledged as a potential method to teach, participants connected the lack of these understandings with lower knowledge and skills in NbSP hence encouraged teaching outside. Even though pre-registration AHP curricula do not stipulate indoor or outdoor teaching for NbSP, AHPs are traditionally taught where most sessions are in person, indoors either in a classroom, simulation or on placement. Educators teaching NbSP could explore the potential of working with community organisations to increase AHP students’ understandings, e.g., through a simulation day where the learning outcomes for the session constructively align with the module content and assessment. This may involve educators reflecting and redesigning session content with and for students. Development of collaborative partnerships with NbSP providers to create a balance of theory and practice examples, potentially with simulation-based learning exercises, may support scaffolding learning for students.

### 4.2 Widening participation and educating for nature connectedness

Limited literature exists on personalizing NbSP classrooms. This includes teaching students online, in person, with flipped learning and in a way to give each student the learning experience they need on this topic area. Personalized curriculum is discussed in the wider literature in the context of medical sciences and some AHPs (e.g., physiotherapists) and concludes it is critical for “*student engagement*” and “*developing critical skills necessary for health-care professionals”* (60). Yet, the Go Zone analysis highlighted the need for teaching students the benefits of NbSP in the context of widening participation (and education) through nature connectedness. One participant suggested *“using multiple methods to present nature connection opportunities*.” This leans toward a personalized approach within the curriculum (60). Reflection workshops for educators on the reduction and removal of barriers to widen participation for people to access NbSP are needed. Information on options for students on how to learn NbSP may support educators to teach practical tips transferable to real-world settings.

### 4.3 Digital flexibility: indoor in person, digital (online/hybrid/VR/mixed reality) or outdoor classrooms

Digital flexibility is required to meet student learning needs, for example reasonable adjustments, childcare arrangements and carer responsibilities. Creating a future ready AHP workforce is the job of educators in HE given AHPs currently work, and will continue to work in telemedicine, in person and hybrid roles. Teaching AHPs complex topics including NbSP is not as simple as indoor, outdoor, in person or online. Students’ “*life situations*” can become the primary influencer of when and where learning a topic is best to occur for them, therefore “*synchronous hybrid”* can provide inclusive personalized education environments (29). In this study, students also highlighted once they choose where, then they explore “*learning preferences*” and social learning groups. Participant results in GCM reflected the wider literature on the potential for changing the teaching environment to reflect the needs of the students first above the topic area. Flexing the classroom to meet the students’ needs requires digital flexibility and can only be created with educators continuing to build their digital skill set.

Participants indicated a preference toward AHPs learning in person, with community organizations in outdoor environments rather than teaching NbSP in virtual or digital environments. Yet, the potential for use of VR and Mixed Reality was acknowledged by participants indicating potential for pedagogic tensions. This coproduced knowledge was dominated by NbSP practitioner and national organization viewpoints with minimal HE educator perspectives. Despite the overarching positive lean in statements toward outdoor teaching in nature, participants acknowledged the logistic and accessibility challenges faced by educators particularly with large groups.

Future exploration of singular stakeholder groups, e.g., AHP educators may illustrate more practicalities for solution focused coproduced teaching. Further research could explore AHP student preferences on learning environment for NbSP alongside which environment provided the most inclusive and effective way for the individuals to learn (and why).

### 4.4 Pedagogical implications

Explicit guidance for AHP educators on how to teach NbSP, what to include, where to teach and how to constructively align the taught session, and meet module learning outcomes requires additional research. Coproducing or codesigning curriculum, often described as best practice across the literature, takes considerable time. Digital flexibility and hybrid teaching has inherent challenges, including the rejection of hybrid teaching by HE itself (28). Educators need the flexibility to teach their students in environments they feel would suit the students’ learning preferences, and ensure reasonable adjustments are met for all students in the session. Use of simulation-based education (31), OSCE stations (30), community mapping assignments and reflective essay or portfolio submission are examples in the literature, echoed by participants, where formative and summative assessment can take place. Simulation-based learning was identified in a meta-analysis covering 145 studies as *“the most effective means to facilitate learning of complex skills across domains*” (31). Constructively aligned curriculum provides part of the structure needed within effective teaching of NbSP, yet this is not always inclusive. Educators, as part of reflective practice, must learn the pedagogical implications for AHP students of ineffective and un-inclusive teaching. The most impactful way to teach NbSP to AHP is not yet fully understood.

Steps can be taken to acknowledge limited educational practices in teaching NbSP and explore inclusive and effective pedagogical practices. One Powerpoint slide on NbSP may illustrate a descriptive version of the topic to students, yet participants in this study argued for the criticality of getting students outside in nature and preferably working with community organizations delivering on the NbSP agenda. Participants offered ways to teach NbSP ranging from indoor nature boxes, digital solutions (including wearable Mixed Reality to experience the sounds and sights of nature or forest bath indoors) to understanding the community classroom environment and student’s needs. Yet, educators face numerous challenges to planning outdoor and off-site classrooms. Meeting the costs of a minibus and hire of a community organization for the day may not meet budget allocations and the financial challenges in the HE sectors. Despite best made plans, weather in the UK is increasingly unpredictable. Extremes of temperatures, as seen with the 2025 heat waves due to the climate crisis can result in challenging environments for students to learn, indoors and outdoors. Participants suggested students need to be present in the environment’s community organizations would normally work in with people who are referred to NbSP. Pressured workloads of educators can severely impact the best pedagogic intentions. Having said this, educators, particularly module and course leaders, have the power to flex the curriculum environment to meet student needs, requiring considerable topic knowledge and teaching skill level. With this flexibility comes educator’s responsibility to constructively align the curriculum alongside existing educational frameworks to ensure quality within HE. Yet, Sreedharan et al. (61) stated “*resources for effective learning and teaching in the allied healthcare domain are limited*.”

Assessment of AHP delivering on the NbSP agenda is not yet underpinned with a clear educational framework. With the rise of AI and increasing potential of students having free access to technology that can write assignments, there is a leaning within HE pedagogical growth to more practical assessments. This includes assessing real world skills used by AHP in a simulation-based education context. This necessitates further research on the specifics of how NbSP could be assessed per AHP group (BSc, MSc, profession-specific CPD). Employing Simulation Based Education, mixed reality and alternative assessment strategies could be the way forward as indicated by participants.

Educators may face students with limited or no understanding of nature connection through choice or life circumstances. *“Compassionate pedagogy”* (62) could be utilised with additional sessions and support for students needing to understand basic principles before advancing through more complex NbSP concepts. The ongoing challenge for educators is to find or make time if students need additional support. Further research is therefore required into appropriate teaching and assessment methods of AHPs that are inclusive, effective and impactful to their future practice.

## 5 Strengths and limitations

### 5.1 Strengths

This seminal article illustrates the first postgraduate study applying GCM to NbSP pedagogy for AHPs. Through transparency of methodology and methods, including attrition, this study has illustrated rigor within an integrative mixed-methods research design. The use of validated, recognized protocols in the GCM software was complemented by understanding the literature supporting this appropriate software. The novel application of GCM to health pedagogy was completed through diverse stakeholder involvement. Participants were from community practice, health, social care, AHP, simulation, and national organization backgrounds. Obtaining stakeholder perspectives from diverse groups like these is challenging. Stoyanov et al. (50) demonstrated rigor through multi-institutional participant sampling, transparent data processing steps and robust stress values reflecting this study. Transparency of data is demonstrated with clear signposting and access to the original data set and analyzed included in this paper (45). GCM as a method provides strong evidence of rigor and trustworthiness given the iterative analysis component and participant engagement (54). This mixed- methods approach leveraged qualitative input (ideas, sorting) and quantitative analysis (multidimensional scaling, cluster analysis and ratings) to enhance analytic rigor and depth. Internal consistency, convergent validity and comparing findings with existing literature and frameworks were all used within the study providing evidence of rigor and trustworthiness.

### 5.2 Limitations

The results from this GCM study are not representative of the whole population of international stakeholders who could contribute to effective teaching and learning of NbSP. Rather, this study represents the initial foundations of coproduction for the purpose of inclusive and effective teaching in this topic area in the UK. This work has potential to feed into national educational and competence framework developments. The limitations of GCM are well known, including variations in expert opinions on the rigor of sample sizes (45). “Cognitive loading” of participants due to the complexity of task within each stage and lack of reporting on excluded data are known limitations (46). Recruitment of participants, workshops on using GCM and data analysis were completed by one researcher. Additional resources may have resulted in separate roles and additional data, e.g., project manager, interviewer, data cleaner.

The sample size is small and potentially unrepresentative of the whole NbSP population. A larger data sample could be pursued through more iterations of recruiting participants. Future research can create more inclusive research spaces, e.g., “taking a written GCM into community settings and NbSP settings to involve community groups and stakeholders with limited access and/or digital skills” (48). An optimal sample size of 20–30 was highlighted in one paper on a pooled analysis of concept mapping studies over 10 years ago (45). This can be interpreted as either per phase of concept mapping (i.e., 20 participants sorting, 20 participants rating) or 20 participants in total across the phases. Although not convenience sampling (i.e., only participants from their university), the lead researcher chose purposive, snowballing sampling through word of mouth, social media and SP networks in the UK.

Other methods and methodologies could be employed: Delphi study (although consensus was not the initial research goal), different types of mixed methods (i. e. increased use of statistically significant language and phrases by participants to reach consensus aligning with pragmatics research). Limited exploration of international pedagogical models was employed for comparison. Future research could explore international pedagogy, stakeholder perspectives and best inclusive and effective practices in teaching NbSP. International sampling and exploration of international pedagogical models for comparison could illustrate pedagogy to be adopted in the UK.

The dual role of the lead researcher (PhD candidate and data analyst) may have influenced findings through selection or interpretation bias (63). Participants may have responded in a way they thought the facilitator wanted them to reply or in a way they wanted their results to be seen. Critical reflection was employed by the lead researcher and discussed in research supervision on the roles and potential challenges including influence on research quality, power dynamics, ethical responsibilities, and the potential for bias (63, 64).

Results are representative of the participants in this study who took part and were identified as completing over 75% of the GCM stages, hence the Go Zone did not include every participant response. Sorting and rating results with less than 75% of answers completed were excluded from the final analysis based on consensus in the field (49). Researcher reflexivity illustrated the challenges of not including or amplifying every participant’s voice by GCM. Reporting of exclusion rates is suggested by some but not all studies for the purpose of transparency in research (46). Results are reflective of the sample at one point in time and could be grown if recruitment occurred over several years. Further research with stakeholders would support deeper understandings of what academics teach, why and in what way when considering reasonable adjustments within future classrooms (65).

Cross-institutional validation of NbSP curricula could be developed collaboratively with the AHP professional bodies and leading educational trainers, e.g., Advance HE as part of pre-registration and CPD standards in future studies. This is fundamental pedagogy for educators to evidence inclusive and effective teaching in all classroom environments.

## 6 Conclusion

This article illustrates results and discussion from the first GCM exercise with U.K. stakeholders in NbSP on how it could be taught in HE to AHPs. This included participant perspectives on what could be included for inclusive and effective classrooms in digital environments. Stakeholder-generated themes illustrated a wide range of topic areas to be included in teaching NbSP. Findings emphasized that “*Experiencing and connecting with nature,” widening participation and deepening reflective learning about nature connection*,” are essential for inclusive and effective teaching practices in NbSP.

The research supports HE providers and regulatory bodies in shaping curriculum and CPD frameworks to prioritize inclusivity, experiential learning and digital adaptability. Recommendations were made by participants on inclusivity, effective teaching and digital environments. These aligned predominately with the need to expose students to nature and nurture their nature connection in a personalized way. This is needed for students to understand and effectively apply NbSP to benefit of their clients. Findings can shape higher education curricula and CPD for AHPs through educator reflection on contemporary content, embracing this opportunity to coproduce inclusive and effective teaching with stakeholders. Pedagogic and digital flexibility, constructive alignment and evaluation of NbSP content and delivery were presented for educator reflection through stakeholders’ lenses.

This study adds to the limited body of knowledge on inclusive and effective teaching of NbSP in digital and in person environments. Further research is required into additional stakeholder perspectives, and to develop actionable strategies for integrating NbSP within national education and professional standards for educators of AHPs on NbSP.

## Data Availability

The original contributions presented in the study are included in the article/supplementary material, further inquiries can be directed to the corresponding author.
